# Long COVID Optimal Health Program (LC-OHP) to Enhance Psychological and Physical Health: Protocol for a Feasibility Randomized Controlled Trial

**DOI:** 10.2196/36673

**Published:** 2022-05-12

**Authors:** Hiyam Al-Jabr, Karen Windle, David R Thompson, Zoe M Jenkins, David J Castle, Chantal F Ski

**Affiliations:** 1 Integrated Care Academy University of Suffolk Ipswich United Kingdom; 2 School of Nursing and Midwifery Queen’s University Belfast Belfast United Kingdom; 3 Mental Health Service St Vincent's Hospital Melbourne Australia; 4 Department of Psychiatry University of Toronto Toronto, ON Canada

**Keywords:** long COVID, COVID-19, optimal health program, telemedicine, integrated care, telehealth, patient care, health intervention, mental health, physical health, psychological health, pandemic, patient support

## Abstract

**Background:**

Long COVID is a collection of symptoms that develop during or following a confirmed or suspected case of COVID-19, which continue for more than 12 weeks. Despite the negative impact of long COVID on people’s lives and functioning, there is no validated treatment or even rehabilitation guidance. What has been recommended thus far is the adoption of holistic management approaches. The Optimal Health Program (OHP) is a brief 5-session, plus booster, psychosocial program designed to support mental and physical well-being that has been used effectively for a range of chronic conditions.

**Objective:**

This study examines the feasibility and acceptability of employing an especially customized version of OHP (long COVID OHP [LC-OHP]) to improve psychological and physical health of people with long COVID.

**Methods:**

This is a feasibility randomized controlled trial that will be running from November 2021 to February 2023. Eligible participants aged 18 years or older who are experiencing symptoms of long COVID will be identified through their secondary practitioners with recruitment to be undertaken by the research team. A total of 60 participants will be randomized into a control (usual care) or an intervention (LC-OHP) group. Outcomes will be feasibility and acceptability of the program (primary); and efficacy of the LC-OHP in improving anxiety, depression, fatigue, self-efficacy, and quality of life (secondary). Up to 20 participants will be interviewed at the end of the trial to explore their experience with the program. Quantitative data will be analyzed using SPSS, and differences between groups will be compared using inferential tests where appropriate. Qualitative data will be transcribed and thematically analyzed to identify common emerging themes.

**Results:**

This is an ongoing study, which began in November 2021.

**Conclusions:**

Long COVID has a significant impact on an individual’s mental and physical functioning. The LC-OHP has a potential to provide people living with long COVID with additional support and to improve self-efficacy. The findings of this study would identify the feasibility of delivering this program to this population and will provide an indication for the program’s effectiveness.

**Trial Registration:**

ISRCTN Registry ISRCTN38746119; https://www.isrctn.com/ISRCTN38746119

**International Registered Report Identifier (IRRID):**

DERR1-10.2196/36673

## Introduction

### Background

COVID-19, as named by the World Health Organization, is a novel virus that emerged in Wuhan, China in December 2019 and spread rapidly across the whole world resulting in a global pandemic [1-3]. From the start of the pandemic to date, there are more than 246 million confirmed cases of COVID-19 across the world, of which there is around a 90% recovery rate, although more than 4 million people have died [4,5]. Several measures have been implemented to limit its spread, including avoidance of public contact, maintaining social distance, case detection, contact tracing, the use of personal protective equipment such as face masks, and encouraging hand hygiene practices [6,7].

COVID-19 infection is generally believed to be a short-term illness, from which people recover in about 2 weeks [8]. Figures indicate that around 80% of affected people develop a mild to moderate disease, and 5% of those with severe disease develop a critical illness [9]. Recovery usually takes 7-10 days following the onset of the mild infection and 3-6 weeks in the severe or critical illness [10]. In some people, certain symptoms persist in the postacute phase [11,12]. This condition has been given several labels, including long COVID, post-COVID syndrome, long haulers, postacute COVID-19, and persistent COVID-19 symptoms [11,13,14]. Whatever the term, each describes an illness in which symptoms persist longer than expected, or where there are lasting effects of the infection [2]. In the absence of an alternative explanation, long COVID encompasses ongoing symptoms of COVID-19 lasting 12 weeks or more [2,12,15-18].

COVID-19 is a new virus; as such, there are many unknown aspects surrounding its trajectory, including who is susceptible to long COVID and why recovery is prolonged for some people. While it is not yet possible to identify who may develop long COVID, there appears to be several risk factors including female sex, increased age [15], presence of more than 5 symptoms in the acute phase [19], associated comorbidities, and extended duration of acute illness [14,19-23].

A recent systematic review, which included 25 studies with 5440 participants, reported the frequency of long COVID to be up to 80% of the included population [17]. Another study showed that some symptoms persisted 3 months post hospital discharge [24]. Moreover, it seems that anyone can develop long COVID, including young people with no preexisting health conditions [18,25]. In the United Kingdom, the Office of the National Statistics and the UK COVID-19 symptom study app show that 1 in 5 people have COVID-19 symptoms that persist after 5 weeks, and 1 in 10 have symptoms that persist longer than 12 weeks [8,26]. To date, more than 8 million cases of COVID-19 have been confirmed in the United Kingdom [5], with more than 2 million people reporting they may have had long COVID [27], but the number is expected to increase as the virus is still active in the community.

Symptoms of long COVID vary between individuals. It seems to affect multiple organ systems in the body, and patients can present with single, multiple, constant, transient, or fluctuating symptoms [12,28], in a continuous or a relapsing and remitting course. According to a recent meta-analysis, the five most commonly reported symptoms are fatigue (58%), headache (44%), attention disorder (27%), hair loss (25%), and dyspnea (24%) [13]. Fatigue was found to be independent of the severity of the initial viral infection and to develop irrespective of a preceding hospitalization [29]. Long COVID represents a challenge for both the patient and the health care provider as it can be difficult to diagnose with certainty and may influence the doctor-patient relationship and trust [30]. Other reported manifestations of long COVID include cough, joint pain, chest pain, and low-grade fever [8,31,32].

Encountered neuropsychiatric symptoms include anosmia, brain fog, and neuropathy [33,34]. COVID-19 doubles the risk of developing a psychiatric disorder [35]. Anxiety, depression, or posttraumatic stress was manifest in 56% of patients with long COVID [36]. Social isolation, decreased physical activity, changed habits, as well as social and economic insecurity that are associated with the pandemic may contribute to developing the physical and psychological symptoms of long COVID [17,37].

The lack of sufficient information on this condition, along with variability in presenting symptoms and disease course between individuals, adds further challenges to identifying the best practice for its management and control. To date, no clear treatment regime is available to mitigate long COVID, and treatments given are dependent on presenting complaints. Minor complaints such as fever or cough can be treated symptomatically with paracetamol, cough suppressants, or antibiotics if a secondary bacterial infection is suspected [14]. Patients are advised to follow the “three P’s principle,” which is as follows: “Pace” to conserve energy when doing daily activities; “Plan” activities across the week; and “Prioritize” necessary tasks to get a mixture of activities that will boost mental health every day [38].

The complexity and diversity of symptoms of long COVID demand the use of individualized care plans [39]. The focus should not be on providing symptom by symptom management, but rather on delivering holistic, integrated care, bringing together patients and health care practitioners from across all specialties to achieve a common goal and meet the long-term needs of this population [11,40,41]. In the United Kingdom, a 5-point plan was recently set by the National Health Service (NHS) in response to the challenge of long COVID [18]. This included the launch of several clinics to tackle the persistent symptoms of COVID-19 [18,31], the creation of “Your COVID Recovery” website that provides reliable and up-to-date information and support [42], and the investment of millions of pounds in research to gain better understanding of this condition. Additionally, guidance was published to support clinicians in providing care to patients with long COVID [12,43].

Attention to mental health is also important as restrictions associated with the pandemic limited the provision of mental health services [44-46]. Although at the beginning, accessing mental health services decreased [47], numbers slowly started to increase and are now at a record high [47-49]. In 2020, the Office of the National Statistics reported that well-being levels were at their lowest since data collection started in 2011 [50], and that this has been greatly influenced by the pandemic. There has been a significantly increased demand on local beds [51], and the need for new or additional mental health support is expected to increase over the next 3 to 5 years [52,53] to a level that is 2 to 3 times that of the current NHS capacity [54]. There is an obvious and critical need to provide alternative supports; integrated, psychological, and mental health support that is readily accessible to all patients.

Long COVID highlights the need for a specialized intervention to manage complex comorbidities. In this context, the Optimal Health Program (OHP) is a form of psychosocial intervention [55] that provides clinicians with a consistent approach to support patient self-management. This approach challenges traditional methods of health care by encouraging patients to be actively involved in their own management [56] as this is anticipated to be more effective than using passive approaches [57]. The OHP has been shown to be beneficial for people with other chronic conditions such as diabetes [58], stroke [59], and chronic kidney disease [60]. However, no research has yet evaluated its use in patients with long COVID. The Long COVID OHP (LC-OHP) program aligns with the NHS and the National Institute for Health and Care Excellence (NICE) guidelines for managing and supporting long COVID by promoting shared decision-making and by delivering care that meets the individual needs and preferences of patients. Additionally, it provides care to patients in an easy-to-understand way and signposts them to useful resources for more support, as recommended by current guidelines [61].

Through the application of the tailored LC-OHP sessions alongside those living with long COVID, this study will examine if this program is feasible and acceptable in improving individual’s psychological and physical health.

### Aim and Objectives

This study aims to examine the feasibility and acceptability of the LC-OHP and evaluate any impact on quality of life, depression and anxiety, fatigue, and self-efficacy in patients with long COVID, compared to usual care. The study objectives are as follows:

#### Primary Objectives

The primary objectives are to determine the feasibility of conducting a 5-week psychosocial intervention in patients with long COVID by identifying the following outcome measures: (1) acceptability, recruitment, and retention rates; (2) participant’s satisfaction with the intervention; and (3) appropriateness of secondary outcome measures.

#### Secondary Objectives

The secondary objectives are to evaluate the preliminary efficacy of the LC-OHP in improving quality of life, depression and anxiety, fatigue, and self-efficacy in people with long COVID, compared to usual care.

## Methods

This protocol is reported using the SPIRIT (Standard Protocol Items: Recommendations for Interventional Trials) reporting guidelines [62].

### Research Design and Setting

This research is a feasibility randomized controlled trial to be conducted from November 2021 to February 2023 by investigators from the University of Suffolk. Potential participants will be identified from a long COVID clinic located within a hospital setting.

### Participants

#### Inclusion Criteria

Patients meeting the following criteria will be included in the study: (1) adults ≥18 years old; (2) COVID-19 infection confirmed through polymerase chain reaction testing or clinical diagnosis from a general practitioner; (3) experiencing post–COVID-19 syndrome (as defined by NICE 2020) 12 weeks or more following onset of symptoms or confirmed through testing; (4) able to participate in a telephone interview in English language (or with accommodated adjustments); and (5) able to consent to participate in the study.

#### Exclusion Criteria

Patients will be excluded if they meet any of the following criteria: (1) children and young adults (17 years and under); (2) unable to consent to participate in study; and (3) unable to participate in a telephone interview in English language.

#### Sample Size

A total of 60 patients will be recruited to the trial, with 30 randomized to the intervention or control group. The sample size has been determined according to the recommendations that at least 30-35 patients be included per group for pilot and feasibility studies [63-66].

### Study Procedures

#### Consent and Recruitment

Eligible participants who are referred to the long COVID clinic will be identified and approached by the clinical team on their initial visit to the clinic that is located at a hospital setting. As part of their initial assessment, the team will provide a brochure that outlines the trial and details of the research team to contact if they would like to take part. After expressing interest, the research team will provide eligible participants with the trial’s participant information leaflet and a consent form with a prepaid envelope. The research team will then contact the participants after 5 working days of sending the documents to answer any queries and to remind them to send their signed consent form.

#### Baseline Data Collection

Following the receipt of signed consent forms, the research team will contact participants to agree on the logistics of completing the questionnaires. Questionnaires will be sent to participants by email or post. With the baseline questionnaire, participants will also complete a short questionnaire to collect demographic data (ie, age, gender, ethnicity, and education). Participants will also have the option to complete the questionnaires on their own (ie, self-completion) or by telephone supported by a member of the research team. Responses to questionnaires will be recorded on a secure electronic database. Two reminders will be sent to nonrespondents. If no response is received, participants will be removed from the study. Table 1 provides a summary of the questionnaires to be completed by participants in the study at baseline, 3 months, and 6 months post randomization.

**Table 1:** Overview of questionnaires.

Questionnaire	Outcome	Details
PHQ-9^a^	Depression	A 9-item validated questionnaire with 4 response options: “not at all” (scored as 0) to “nearly every day” (scored as 3). PHQ-9 total scores range from 0 to 27, with scores of ≥5, ≥10, and ≥15, representing mild, moderate, and severe levels of depression severity [67]. The questionnaire can be administered over the phone.
GAD-7^b^	Anxiety	A 7-item, easy-to-use, self-administered questionnaire, answered using a 4-point scale (from 0 to 3). It is used as a screening tool and severity measure for generalized anxiety disorder [68,69].
GSE^c^	Self-efficacy	A 10-item questionnaire rated using a 4-point Likert scale (“not at all true” to “exactly true”). The questionnaire tests the individual’s self-efficacy (ie, his or her ability to organize and execute certain behaviors that are necessary in order to produce given attainments). Higher scores are indicative of higher self-efficacy. The questionnaire is valid and reliable with high internal consistency [70].
EQ-5D-5L^d^	Quality of life	A validated 5-item questionnaire scored on 5-item answers. It provides a generic measure on the following health dimensions: mobility, self-care, usual activities, pain or discomfort, and anxiety or depression [71]. The questionnaire also includes a visual analogue scale with scores ranging from 0 to 100 to reflect current health status [72].
FAS^e^	Fatigue	A validated 10-item questionnaire that is answered using a 5-point answer scale: “never” (scored as 1) to “always” (scored as 5) [73,74].

^a^PHQ: Patient Health Questionnaire.

^b^GAD-7: Generalized Anxiety Disorder.

^c^GSE: General Self-Efficacy.

^d^EQ-5D-5L: European Quality of Life 5 dimensions, 5 levels.

^e^FAS: Fatigue Assessment Scale.

### Randomization, Allocation, and Blinding

Participants will be randomized following the receipt of baseline data to either intervention or control group using a computer-generated block randomization. To avoid bias, an independent person will carry out participant randomization. Due to the nature and length of the intervention, it is not possible to blind either the research team or the participant to the treatment allocation.

Participants allocated to the control group will receive usual care provided to patients with long COVID, depending on presenting complaints and assessments. The intervention group will receive the LC-OHP plus usual care.

### Intervention

The OHP is a person-centered model that focuses on health as defined by patients. It aims to support people with mental or physical illness by using a collaborative therapy–based self-efficacy intervention [55,75-78]. The program addresses psychological and physical dimensions of health [60], is flexible, and can be delivered by a range of practitioners. It can also be delivered at all stages of the care trajectory; in inpatient and outpatient settings, at homes, or by video conferencing, and to groups or to individual patients [77]. By enhancing self-efficacy and self-management skills, the program works on shifting the focus of an individual’s illness from being “dependent on services” to being “supported by services” [78], which is thus anticipated to reduce pressure and financial demands on health care systems.

The OHP can be delivered in 8- or 5-session sequential formats, with both types of delivery including a follow-up “booster session” [55] [77-80]. It offers its users (ie, facilitators and participants) a written booklet that includes different skills and information to support self-efficacy, besides regular reviews of previous sessions to follow progress [55]. In this trial, recognizing that fatigue is a core component, the LC-OHP will be delivered in 5 sequential sessions. The program’s key elements are summarized in Table 2.

**Table 2:** LC-OHP^a^ sessions.

Session	Title	Content
1	Optimal health	What is optimal health? Optimal health wheel
2	I-Can-Do-Model	Strengths and vulnerabilities Stressors and strategies Health plans 1 and 2
3	Factors of well-being	Medication and metabolic monitoring Collaborative partners and strategies Health Plan 3
4	Visioning and goal setting	Defining change Orientation and preparation Creative problem-solving and goal setting Reflection and celebration
5	Building health plans	Health plans 1, 2, and 3 My health journal
Booster	Reflecting on the learning in the transformational journey to sustain well being	Reflecting on learning in the transformational journey to sustain well-being

^a^LC-OHP: Long COVID Optimal Health Program.

Sessions will be delivered weekly with a booster session to be held 3 months after the last session. The booster session will target reviewing health plans and reflecting on achievements made toward health-related goals. Sessions will be held either in a 1:1 encounter with the facilitator, or in groups, or using a mixture of both, depending on participants’ preferences. Each 1:1 session will last up to one hour, with breaks provided upon participant’s request, and if necessary, the session can be completed at another time. Group sessions will last up to 90 min, and breaks will be provided upon request. Sessions will be held online (using a convenient platform) or by telephone (to those who do not have access to online facilities). Sessions will be audio-recorded to be checked by another member of the research team to confirm fidelity of program delivery. Participants can contact facilitators between the sessions if they have any questions or wish to change the time for the next session.

Facilitators have received prior training in delivering the program during a 2-day workshop delivered by one of the investigators (DJC) who developed the OHP program. Regular supervision will also be provided to facilitators to ensure fidelity with program delivery and to discuss any concerns. Additionally, a fidelity checklist will be completed by the facilitator after the end of each session to ensure all core components of each session have been covered. The checklist will also be reviewed by another member of the research team to ensure fidelity.

### Quantitative Data Collection

Following participation and baseline data collection, the research team will contact participants at 3-month intervals after randomization at a prearranged time to complete the questionnaires detailed in Table 1. At the end of the trial, participants in the intervention group will be asked to evaluate the LC-OHP by completing the Course Experience Questionnaire.

### Qualitative Interviews

After the end of the trial, participants allocated to the intervention group will be invited to participate in a semistructured telephone interview conducted by an independent researcher (not the OHP facilitator). The interview will target exploring participants’ experience with the LC-OHP, identify their views on the program, and share any suggestions for improving the program for the future. Interviews will be audio-recorded and will be conducted at a time convenient to the participant. Interview duration will last up to 30 min (a max of 40 min if a participant requests more time) and will be guided by a topic guide developed by the research team. Participants will be interviewed until data saturation is reached. Textbox 1 shows a summary of the interview topic guide list.

**Summary of the interview topic guide list.**

**Main questions**

- Can you please tell me a little bit about why you agreed to take part in the study?
- Describe your experience of being involved in the program.
- What do you think about the materials and support provided throughout the program?
- Do you feel that there may be changes that could be made to the program? What, how, and why?

### Internal Pilot

**Figure 1:**
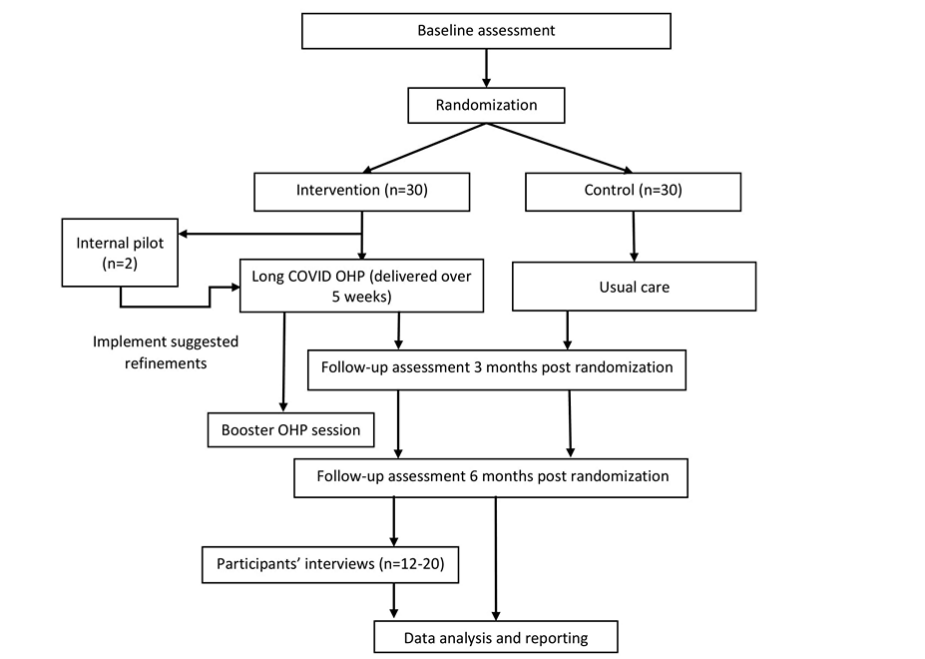
Participants' flow in the study. OHP: Optimal Health Program.

The trial will start with an internal pilot, with the first 2 participants receiving a 1:1 session delivery. These participants will have a short interview (10 min) at the beginning of each session by the facilitator to identify their views about the previous session with respect to the program’s materials and to inform any necessary refinements before starting the program with other participants. The program will start 2 weeks following the internal pilot in order to give enough time for any modifications to be implemented. Figure 1 presents a flowchart of the trial process.

### Participant Withdrawal

Participants can leave the study at any time without their care being compromised. All data collected from participants prior to withdrawal will be included in the analysis.

Participants’ withdrawals will be noted, and reasons will be classified under the following categories: (1) illness or death; (2) dropout or relocation; (3) loss of contact; and (4) failure to return the questionnaire.

Additionally, for the purpose of ensuring fidelity, the trial’s research team will interview a sample of participants who withdrew from the intervention to identify reasons behind their withdrawal, and to collect any suggestions that may improve the program for future participants. The withdrawing participants will be free to decline this interview without their usual care being affected.

### Data Analysis

Quantitative data analysis will be performed using SPSS (IBM Corp). Descriptive data will be presented using means, interquartile range, and percentages. Differences between the groups will be compared using appropriate inferential tests.

For the qualitative data, interviews will be recorded (with informed consent), transcribed verbatim, coded, and thematically analyzed (using an inductive thematic analysis approach) to identify common emerging themes [81]. Transcripts will be continuously revisited, and the accuracy will be verified by listening to the recordings and comparing them with the transcripts. Coding of data will be conducted using NVivo (QSR International), and codes will be checked by another member of the research team to ensure appropriate and consistent coding process. Any disagreements will be resolved by consensus, and by referring to the transcripts and original recordings.

### Data Management and Monitoring

All data collected from participants will remain strictly confidential, and all participants will be pseudonymized and coded with a study number. Data will be securely stored at a central computer drive accessible only to members of the research team. Principles of the General Data Protection Regulation 2018 will be followed with respect to data storage, processing, and destruction. Any paper documents will be stored in a securely locked filing cabinet located at the Integrated Care Academy at the University of Suffolk, accessible only to members of the research team.

A Data Management Committee (DMC) is set for the trial and will include the sponsor, 2 clinicians, 2 independent researchers, and 2 members of the public. The committee will meet at least 3 times (at the start, middle, and end of the trial) and as needed. The DMC will oversee all aspects of the study and will monitor the trial’s progress and deadlines adherence, ensuring participant safety is maintained and agreeing on any amendments to the protocol. Any amendments conducted will be notified to ethical committees.

The trial will be prematurely terminated if severe and unacceptable safety risks occur. Adverse events will include any unfavorable and unintended events experienced by a participant during the study that are reported by the participant or observed by the investigator or medical staff. Serious adverse events will be any untoward medical occurrence that could result in death, hospitalization or prolongation of existing hospitalization, persistent or significant disability or incapacity, or a congenital anomaly or birth defect. Adverse events and serious adverse events will be registered and presented to the DMC to be reviewed. The DMC will have the power to stop the trial on safety grounds. However, it is highly unlikely for early termination as the OHP has been previously used with people with chronic medical conditions and has been proven effective, without untoward outcomes.

### Patient and Public Involvement

The OHP was tailored to long COVID with input from the long COVID clinic clinicians (2 occupational therapists). In addition, guidance was provided on the mode and timing of questionnaire administration with changes made to processes of administration to ensure participants will be prepared and supported. Mindful that the most common symptom of long COVID is fatigue and brain fog, and along with the overall focus of this study (ie, assessing the feasibility and acceptability of LC-OHP), patients with long COVID were not involved in refining the OHP at this stage. However, feedback from patients who used this program in previous studies has been implemented to make the program more succinct, colorful, and visual. Additionally, the delivery of the LC-OHP sessions will be arranged at patients’ convenience in terms of time and mode of delivery. Moreover, the views of patient participants who receive the program sessions will be collected by interviewing the pilot participants and the intervention participants at the end of the trial to further implement and adapt the program to people with long COVID. Finally, members of the public will form part of the trial DMC to monitor the progress of the study and contribute to the dissemination of its findings.

### Ethics Approval

This study received approval from the University of Suffolk Ethics committee (RETH21/004) and from the NHS Health Research Authority (IRAS 304234).

The study will be conducted in accordance with the Health Research Authority and the University of Suffolk guidelines on ethical conduct in research, as well as the approved study protocol.

### Dissemination Plan

The findings of this study will be communicated using different dissemination pathways. These include the use of academic pathways (ie, peer-reviewed journal articles and abstract submissions to local, national, and international conferences), social media pathways (ie, Twitter, YouTube, and LinkedIn), and sending summary findings to participants taking part in this study. The research team will work closely with the DMC, which will include members of the public to identify other appropriate pathways to disseminate the findings to the wider population.

## Results

This is an ongoing study, which began in November 2021. The outcomes will be feasibility and acceptability of the program (primary); and its efficacy on improving anxiety, depression, fatigue, self-efficacy, and quality of life (secondary). It is hypothesized that the LC-OHP will be acceptable by participants, will improve their health and psychosocial functioning, and will enable them to gain better understanding of this condition, increasing patient self-efficacy of their symptoms.

## Discussion

### Principal Findings

In moving this program forward, the feasibility and acceptability of the LC-OHP will be tested along with early measures as to its effectiveness. It is widely acknowledged that long COVID is a chronic condition that is associated with long term, multisystem manifestations. It is a serious health problem that has a significant impact on an individual’s physical, social, and psychological functioning. Though there is a good deal of uncertainty and much to be learned about long COVID, there is a pressing need for a specialized intervention to manage the condition.

While the LC-OHP has been used effectively for previous other chronic conditions [58-60,77], it has yet to be used for long COVID. This study has been designed to apply this program to people living with long COVID; however, there is no prior evidence to rely on at present as it is well recognized that COVID and long COVID are new conditions, and researchers and clinicians all over the world are still assessing how best these can be treated. Thus, the program in this study is going to be one of the first and innovative approaches to be used in order to provide additional support to this group of people.

The use of the OHP within this feasibility study offers a holistic approach that can provide a pathway for people to aid them through their recovery and to encourage them to be actively involved in their own management. It uses a person-centered approach and is in line with NICE guidelines and with the NHS plan 2021-22 for managing people with long COVID. It is therefore anticipated that an OHP specifically tailored to patients with long COVID will be acceptable by participants, will have a high retention rate, will improve their health and psychosocial functioning, and will enable them to gain better understanding of this condition, increasing self-management of their symptoms. This increase in self-management may reduce individuals’ contact with health or care services, reducing the demand on the NHS and care services seen during the pandemic. The impact of COVID-19 on mental health is widely acknowledged, and the LC-OHP could be a suitable approach to mitigate this impact and increase individuals’ empowerment and independence. Moreover, understanding participants’ views and perceptions on the LC-OHP is hoped to improve the program to provide better support for patients with long COVID.

### Strengths and Limitations

The study has some limitations. Due to the nature of the intervention, participants and researchers will not be blinded to group allocation; however, to avoid researcher-induced bias, randomization and interviews will be conducted independently.

To the authors’ best knowledge, this is the first study to examine an individualized holistic psychosocial program designed to address the needs of adults experiencing long COVID. The study is anticipated to provide evidence for the feasibility and acceptability of the used intervention in improving health outcomes for people experiencing long COVID. This feasibility trial uses a mixed methods design to assess procedural and methodological data in preparation for a fully powered large-scale trial.

### Future Directions

The findings of this study will be used in implementing the suggestions given by participants in further adapting and tailoring the LC-OHP program to make it more suitable to people living with long COVID. The findings will also be used as pilot data to support conducting a fully powered large-scale randomized controlled trial to recruit a higher number of participants from multiple sites and from different geographical areas.

## Abbreviations

DMC: Data Management Committee
LC-OHP: Long COVID Optimal Health Program
NHS: National Health Service
NICE: National Institute for Health and Care Excellence
OHP: Optimal Health Program
SPIRIT: Standard Protocol Items: Recommendations for Interventional Trials
